# COVID-19 Containment Measures at Childcare and Schools in 19 European Countries: An Observational Study on Local, Federal and National Policies

**DOI:** 10.3389/ijph.2021.1604010

**Published:** 2021-04-21

**Authors:** Danielle E. M. C. Jansen, Johanna P. M. Vervoort, Károly E. Illy, Adamos Hadjipanayis

**Affiliations:** ^1^ Department of General Practice, University of Groningen, University Medical Center Groningen, Groningen, Netherlands; ^2^ Department of Sociology, University of Groningen, Groningen, Netherlands; ^3^ Department of Health Sciences, University of Groningen, University Medical Center Groningen, Groningen, Netherlands; ^4^ Department of Pediatrics, Hospital Rivierenland, Tiel, Netherlands; ^5^ Dutch Paediatric Society, Utrecht, Netherlands; ^6^ Medical School, European University Cyprus, Nicosia, Cyprus; ^7^ European Academy of Paediatrics (EAP), Brussels, Belgium

**Keywords:** children, adolescents, Europe, COVID-19, containment measures, policy

## Abstract

**Objectives:** After childcare and schools have been closed in March 2020 to prevent the spread of COVID-19, they were open again in most European countries after the summer holidays till early autumn. Aim of this study is to give an overview and to compare COVID-19 childcare and school containment policies in 19 European countries.

**Methods:** We collected data on containment measures among delegates of the European Academy of Pediatrics (EAP), through an online, closed questionnaire in the second half of October 2020.

**Results:** Most policy has been formulated for secondary education. In all three settings policy was most often formulated for individual hygiene, cleaning of surfaces, exclusion of sick children, ventilation, distance between children and between children and teachers. In secondary schools, policy is formulated on face masks in and outside the class. School closure, cancellation of physical education and class size reduction are measures for which the fewest countries have formulated national policies.

**Conclusion:** We recommend to accompany the opening of children’s facilities and schools by surveillance studies that further clarify questions about control measures implemented to halt COVID-19 pandemic.

## Introduction

After childcare and schools have been closed in March 2020 to prevent the spread of COVID-19, they were open again in most European countries after the summer holidays till early autumn (1). The rationale behind reopening schools is threefold: firstly, there is little and inconsistent evidence of transmission from children to adults. There is some evidence suggesting that youth between the age of 10–19 years spread COVID-19 to the same extent as adults (2); however, other studies report little evidence of transmission from children to adults, which might suggest that (younger) children do not appear to contribute significantly to the spread of COVID-19 (3–6). Secondly, major concerns were raised by professionals dealing with children and adolescents that school closures were doing more harm than good because it might lead to several adverse health consequences in children and adolescents such as an increase in mental health problems and weight gain due to unhealthy diet and little exercise (7, 8). Thirdly, children have the right to education (9). But although it seems that the spread of COVID-19 from children to adults is less common, children do have the potential to play a role in community transmission, precisely because the large number of contacts they have in settings such as childcare centers and schools (10). Therefore, there should be a well-considered balance between the possibilities for children and adolescents to enjoy education and the risk that they pose for spreading the virus to adults (i.e. teachers and parents) (11).

Although there is nationwide agreement on the importance of school for children and adolescents, there is a lot of discussion between and within countries about the measures to take when reopening schools. Policy decisions regarding COVID-19 containment measures at schools are very challenging given the contradictory evidence how to act so that it is safe enough for children and teachers to return to school. In addition, for some of the control measures, for example wearing masks, the question is whether the advantages outweigh the disadvantages. Moreover, schools differ greatly from each other—for example regarding culture and pupils’ as well as teachers’ age, so that an unambiguous policy decision regarding the use of face masks in classrooms may not be feasible (12).

The inadequacy and contradiction of evidence about the effectiveness of COVID-19 containment measures at schools, is reflected in a diversity of policies both within countries and also across countries (13). A summary of school re-opening models by 15 countries (14) shows that in July 2020, most models of school re-opening involved reductions of class size, increasing physical distance between students, and keeping students in defined groups with limited interaction between groups to reduce the potential for wide-scale transmission within schools. In addition, a number of countries used alternate shifts (morning, afternoon) or alternate days or had re-opened schools only for younger or, to a lesser extent, older students in order to accommodate the increase in resources (classroom space, teachers, etc.) required for smaller class sizes. In a number of countries, face masks were required for students and/or staff in schools. Systematic school-based testing for SARS-CoV-2 virus or antibodies is being done on a small scale in a limited number of settings (14).

Now that we are several months further in the corona crisis and we, through trial and error, know more about the feasibility and effects of COVID-19 containment measures at schools, an updated overview of these measures is desirable. The aim of this study is to give an overview and to compare COVID-19 childcare and school containment policies in 19 European countries. This overview might trigger countries to compare and, whether or not, adjust their own policy plans in this area and it might inspire future practice and research.

## Methods

### Study Design

We performed an observational study in the second half of October 2020.

### Setting and Participants

We recruited respondents from the European Academy of Pediatrics (EAP) network. The EAP exists to promote the health of children and young people in Europe. It aims to improve standards in training, service and research and to represent the professional interests of paediatricians in the EU. We sent our request to fill in the questionnaire via email at October 14, 2020 to EAP-delegates of 43 European full member countries + Israel (https://www.eapaediatrics.eu/about/members/). We sent a reminder on October 26, 2020 through mail and the survey was closed on 9th November. Delegates of 19 of the 44 countries answered the questionnaire; we received data of 43% of the countries. We did not ask for reasons for non-response. However, representatives from two countries indicated they did not complete the questionnaire because of this policy changing so quickly. All responded in the second half of October 2020.

### Measures

Respondents were requested to fill in a questionnaire on federal and national infection control measures in childcare facilities (care and education services for children before school age) and primary and secondary schools in Europe for the prevention of SARS-CoV-2 transmission. Respondents could fill in an online questionnaire or a questionnaire in Word-format. The questionnaire comprised 42, mostly closed, questions divided into four parts. The first part included questions to collect background information of the participants (profession, country and date of completion of questionnaire). The second, third and fourth part was focused on inquiring 13 infection control measures in respectively childcare, primary and secondary school: if there is a policy (yes, no, sometimes or in some cases), and, if yes, sometimes or in some cases, if the control measures are national policy or policy that differs per state, and if the control measures are mandatory or not.

### Analysis

**FIGURE 1:**
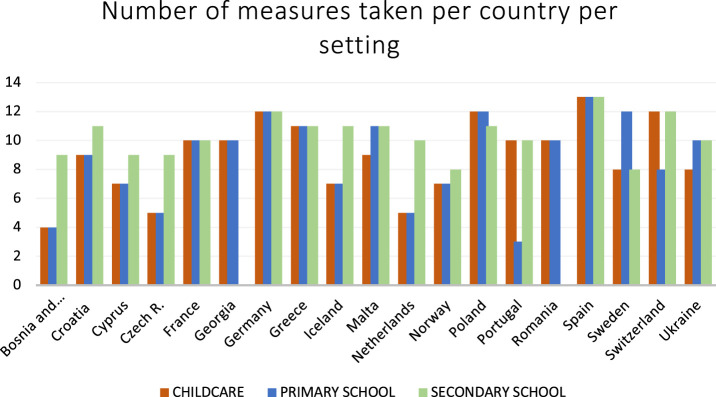
Total number of measures taken (in all cases, in some cases and sometimes) per country in childcare, primary school and secondary school (*19 European countries, 2020*)*.* *Missing data from Georgia and Romania for Secondary School.

The absolute (N) frequencies of all the answers have been calculated and are shown in the tables. To provide a comprehensive overview of all the answers in one table, answers were color coded (green = policy, red = no policy, yellow = policy in some cases or sometimes), supplemented with text indicating if policy is national or state and mandatory or not mandatory. Some countries had multiple participants responding, therefor answers between those respondents were compared. If the answers of respondents of the same country were incongruent, the measurement was not included in the table (Figure 1) and was categorized as ‘unknown’ (Tables 1–3). An answer was categorized as missing if the question was not answered by the respondent.

**TABLE 1:** Country of residence of the participants, date on which respondents completed the survey and the weekly change in COVID-19 infections (regarding the week in which the questionnaire was completed) (*19 European countries, 2020*).

Country of residence	Date of completion the survey	Weekly change in COVID-19 infections in absolute numbers and %^c^ (15)
Bosnia and hercegovina/Rep. Of srpska	October 27, 2020	+4.135/+66,73%
Croatia	October 20, 2020^b^	+4.369/+90,47%
	October 26, 2020	+6159/+66,96%
Cyprus	October 15, 2020	+218/+124,57%
Czech republic^a^	October 15, 2020^b^	+21.418/+64,12%
	October 16, 2020	
France	October 27, 2020	+58.069/+26,66%
Georgia	October 19, 2020	+5.271/+92,75%
Germany	October 27, 2020	+36.542/+54,37%
Greece	October 15, 2020	+389/+15,78%
Iceland	October 15, 2020^b^	−50/-8.5%
	October 26, 2020	+75/+18,94%
Malta	October 16, 2020	+59/+10,89%
Netherlands	October 19, 2020^b^	+9.223/+17,83%
	October 19, 2020	
Norway	October 15, 2020	−157/-14,65%
Poland	October 31, 2020	+46.069/+61,66%
Portugal	October 26, 2020	+7.116/+39,42%
Romania	October 14, 2020	+5.727/+31,23%
Spain	October 15, 2020	+11.853/+16.31%
Sweden	October 15, 2020	+1.187/+27,78%
Switzerland	October 16, 2020	+8.030/+134,75%
Ukraine	October 18, 2020	+3.264/+9.44%

^a^ Primary and secondary schools closed on October 14th, childcare remained open.

^b^ Dates on which respectively the first and the second respondent completed the survey.

^c^ Weekly change is reported regarding the week in which the questionnaire was completed.

**TABLE 2:** Overview of COVID-19 containment measures in childcare per country: if there is a policy (green), a policy in some cases or sometimes (yellow), if there is no policy (red), if it is unknow if there is a policy (grey) or when multiple respondents answered but the answers didn’t match (blue) (19 European countries, 2020).

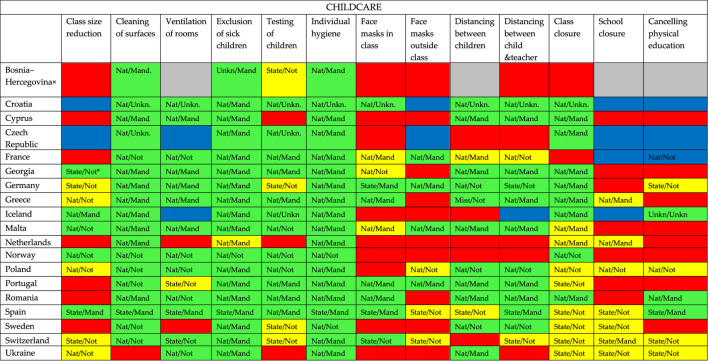

^a^ Nat = national policy; State = state policy; Mand = mandatory policy; Not = not mandatory policy; Unkn/ = unknown if it is national policy; /Unkn = unknown if the policy is mandatory.

× = Bosnia and Hercegovina/Rep. of Srpska.

**TABLE 3:** Overview of COVID-19 containment measures in primary school per country: if there is a policy (green), a policy in some cases or sometimes (yellow), if there is no policy (red), if it is unknow if there is a policy (grey) or when multiple respondents answered but the answers didn’t match (blue) (19 European countries, 2020).

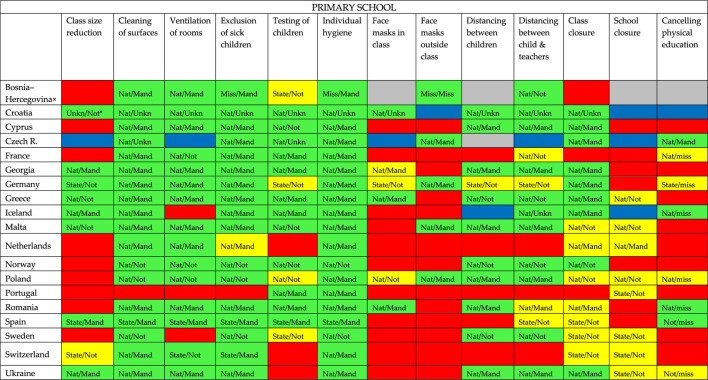

^a^ Nat = national policy; State = state policy; Mand = mandatory policy; Not = not mandatory policy; Unkn/ = unknown if it is national policy; /Unkn = unknown if the policy is mandatory; Miss = missing data on whether the policy is national or federal policy and/or missing data on whether or not the policy is mandatory.

× = Bosnia and Hercegovina/Rep. of Srpska.

## Results

The questionnaires were filled in between 14 October and October 31, 2020 (Table 4). All respondents are paediatricians; some are also president of the national pediatrics association, scientific researcher or public health experts or a combination of multiple of those functions.

**TABLE 4:** Overview of COVID-19 containment measures in secondary school per country: if there is a policy (green), a policy in some cases or sometimes (yellow), if there is no policy (red), if it is unknow if there is a policy (grey) or when multiple respondents answered but the answers didn’t match (blue) (19 European countries, 2020).

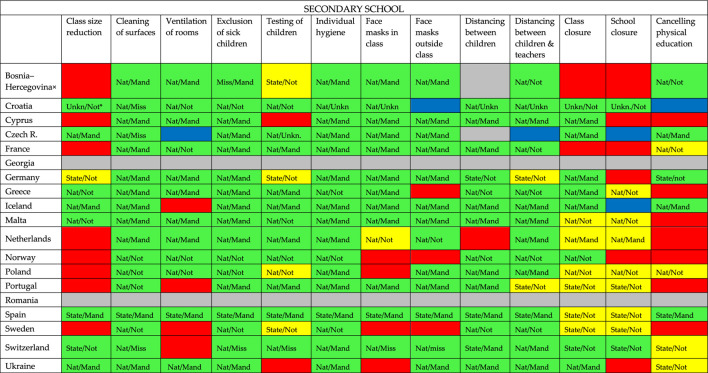

^a^ Nat = national policy; State = state policy; Mand = mandatory policy; Not = not mandatory policy; Unkn/ = unknown if it is national policy; /Unkn = unknown if the policy is mandatory; Miss = missing data on whether the policy is national or federal policy and/or missing data on whether or not the policy is mandatory.

× = Bosnia and Hercegovina/Rep. of Srpska.

Figure 1 shows that - within the three settings–we see clear differences between countries. France, Germany, Greece, Poland and Spain are the countries that have implemented the most COVID-19 containment measures in both childcare, primary school and secondary school, followed by Croatia, Cyprus, Iceland, Malta, Sweden, Switzerland and Ukraine. The other countries have significantly fewer measures within one (Portugal) or the majority of these settings (Bosnia and Hercegovina/Rep. of Srpska, Czech Republic, the Netherlands and Norway). Georgia and Romania have implemented many measures in childcare and primary school, but information on secondary school is missing.

Tables 1–3 show us the extent to which there is policy in different settings: Table 3 clearly shows that–compared to childcare and primary school–more policy has been formulated in all cases (instead of sometimes or in some cases) for secondary education.

Tables 1–3 show that almost all participating European countries show more or less similar policy in childcare, primary and secondary schools regarding individual hygiene, cleaning of surfaces, and exclusion of sick children.

Individual hygiene is national or federal (in case of Spain) policy in all three settings in every responding country and in most countries mandatory (except for Norway and Sweden (all settings) and Greece (not mandatory in secondary school)). Cleaning surfaces is national or federal (in case of Spain) policy in all three settings in every country except for Ukraine (childcare) and Portugal (primary school). This policy is mandatory in most countries. In all countries, except for the Netherlands and Portugal, it is policy to exclude sick children from childcare and/or primary school to prevent the spread of COVID-19. In the Netherlands sick children are not excluded from childcare and primary school in case of mild, cold-like symptoms, such as a runny nose and a cough. If symptoms are more severe and include fever, then they are excluded. In all responding countries it is policy to exclude sick children from secondary school. Closure of childcare and primary school is in most countries no policy; if it is policy, it is sometimes or in some cases and in most countries not mandatory, it often differs per state. Regarding secondary schools, only the respondents of Croatia and Switzerland indicate having a policy. In addition, seven countries seem to have a policy in some cases/sometimes of which only in the Netherlands this policy is mandatory.

### Childcare

Regarding the other COVID-19 containment measures differences between the countries are seen: ventilation of rooms, testing of children, distancing between children and between children and teachers, and class closure are containment measures—albeit to a lesser extent than individual hygiene, exclusion of sick children and cleaning of surfaces—that are still policy in the majority of countries. Face masks in and outside class, class size reduction, and canceling physical education are the containment measures for which many respondents indicate that there is no policy or a policy for some cases/sometimes.

### Primary School

In primary schools, ventilation of class rooms, testing of children, distancing between children and between children and teacher, and class closure are containment measures that is often national or state policy in the participating countries. The remaining measures (class size reduction, face masks in and outside class and canceling physical education) is in the majority of the participating countries no policy in primary schools. In some countries, school closure is only national or state policy sometimes or in some cases (Greece, Malta, the Netherlands, Poland, Portugal, Sweden, Switzerland and Ukraine).

With the exception of individual hygiene, exclusion of sick children and cleaning of surfaces, there is variation in the policy obligation. Only with regard to wearing a face mask outside the classroom are all responding countries in line with the obligation of this policy.

### Secondary School

In secondary education, distancing between children and between children and teachers, face masks in and outside the class, ventilation of rooms, testing of children and class closure is in most of the countries national policy and in most of the countries mandatory policy (except for testing of children and class closure: that policy is in slightly more countries not mandatory than mandatory). Class size reduction is a measure for which far fewer countries have formulated policy. This also applies to canceling physical education.

## Discussion

Our study provides a European overview of COVID-19 containment measures at schools. The results show that–compared to childcare and primary school–more policy has been formulated in all cases (instead of sometimes or in some cases) for secondary education. The measures for which policy is most often formulated do not differ much between childcare, primary and secondary school: in all three settings policy (for all cases) is most often formulated for individual hygiene, cleaning of surfaces, exclusion of sick children, ventilation, distance between children and distance between children and teachers. In secondary schools, additional policy is formulated on face masks in and outside the class. School closure, cancellation of physical education and class size reduction are measures for which the fewest countries have formulated national policies.

Although we can show similarities in COVID-19 containment policies between countries, our study also shows that there are differences between countries. There are countries that have taken many measures, but there are also countries, such as Bosnia and Hercegovina/Rep. of Srpska, Czech Republic, the Netherlands and Norway, where relatively few measures have been taken. There is also variety in the degree of obligation of policy and whether the policy always applies or only in some cases. Are some countries taking more measures than other countries because of the worrying figures? Or are these countries taking the measures to avoid worrying figures? Comparisons of the number of COVID-19 containment measures on childcare or schools per country and the COVID-19 situation at the time of completing the questionnaire do not reveal an unambiguous picture. For example, countries that take many containment measures in childcare and schools concern both countries where a small to medium increase is taking place (for example Germany, Greece, Poland and Spain) and countries where a large increase (for example Cyprus and Switzerland in the number of confirmed COVID-19 infections can be seen. This also applies to the countries in which the least COVID-19 containment measures are taken: in Bosnia and Hercegovina/Rep. of Srpska and the Czech Republic a medium increase in confirmed COVID-19 cases was seen and in the Netherlands, the number of confirmed COVID-19 infections was increasing fairly slow; in Norway it was decreasing.

Our study cannot lead to conclusions that certain childcare and school COVID-19 containment measures lead to a change in confirmed COVID-19 infections. After all, in addition to school containment policies, there is other policy in the community that might be responsible for an increase or decrease in the amount of COVID-19 infections. The uncertainty we still face is the inconclusiveness of evidence on the magnitude of the transmission of SARS-CoV-2 by children and adolescents. In addition, there are more and more studies showing that infections do not take place at school that much, but much more in society outside of school and that infection rates in schools reflect infection rates in the region where the school is located (16, 17).

Our results show that there is diversity in measures that can have a significant impact on the schooling of children and adolescents. For example, distancing between children and between children and teachers, class closures, class size reduction, and face masks in and outside classrooms. This variety probably has to do with increasing or decreasing figures, but also with the possibilities available to properly implement the measures. How feasible is it for example for schools to ensure that pupils keep their distance from one another?

### Strengths and Limitations

Our study findings are limited to 19 countries. Our conclusions might not be representative for the other European countries. A limitation is that the data presented are based on the report of EAP-delegates, who based their answers on national statistics, available documents and possibly on the input of colleagues in their country. In many cases the answers were not accompanied by supporting documents by the participating paediatricians, so some answers might be based on the opinion of the paediatricians themselves. This may have commenced some bias, as the delegates may have varied in the degree of inquiry. Another limitation is that we did not provide more information on the containment measures such as start- and end date of the containment measures or why or in which cases preventive measures were only applied partially or in certain situations. Although we considered collecting these data, we decided to not do this because we thought these questions required too much effort from the respondents to answer them reliably. In addition, COVID-19 school containment policy is highly reactive to and severely dependent on the development of the number of infected people per country. At time of answering the questionnaire it was mostly not clear for example how long a certain policy had to be continued. These limitations do not threaten in our view the main conclusion of our study, which is that there are differences in COVID-19 school containment policies between countries without knowing what the rationale behind most of the policies is.

### Implications

With regard to the COVID-19 measures it is essential to find out how this affects children and adolescents in general, and especially the children who are vulnerable. Research shows that the COVID-19 measures specifically have an influence on this group: children who are already struggling because for example they are growing up in poverty, suffer a greater educational disadvantage as a result of some COVID-19 measures than children who are better off (18–20). We have to be sure that the sometimes rigorous measures in settings where children spend a large part of their day are more effective than they create disadvantages. That is not the case yet; we have insufficient insight into the effectiveness and consequences of measures. Evidence on the use on for example non-medical face masks is ambiguous: while some studies state that evidence is scarce and of very low certainty, (21) other studies do show that public mask wearing could be effective (22) but that there is a need to understand how masks can be used throughout the day, for example by children at school (23). A recent systematic review concludes that current policies of at least 1 m physical distancing are associated with a large reduction in infection (22). However, only few studies assessed the effect of interventions in non-health-care settings. Although beneficial associations were seen across settings, it is unclear to what extent the benefits of the public health measures also apply to children at school.

Because of the ambiguous results, the opening of schools and children’s facilities should be accompanied by well structured, model surveillance studies that further clarify outstanding questions about the hygiene control measures implemented to effectively halt COVID-19 pandemic. At the same time, detailed analysis of the chain of infection is of utmost importance in order to evaluate the control measures in place but also for infection control. The above steps are crucial in evaluating and verifying the effectiveness of the required hygiene measures.

### Conclusion

In our study we present the COVID-19 policies and measures of 19 European countries across three different settings. We show to what extent this policy is mandatory and the extent to which the policy applies. Our conclusion is that there is are differences between countries while–due to the inconclusiveness of evidence on the magnitude of the transmission of SARS-CoV-2 by children and adolescents–we still do not know what the rationale behind most of the policies is.

Children and adolescents are our future. The diversity in COVID-19 school containment policy shows that there is no unambiguous European vision of the policy. However, we should be very careful on applying measures to children or adolescents of which we do not know the effectiveness, but of which we do know that it can be detrimental to certain groups, for example for those who are vulnerable. Research shows that the COVID-19 measures specifically have an influence on children who are already struggling because for example they are growing up in poverty; they suffer a greater educational disadvantage as a result of some COVID-19 measures than children who are better off.
